# Effect of Nitrate Leaching Caused by Swine Manure Application in Fields of the Yellow River Irrigation Zone of Ningxia, China

**DOI:** 10.1038/s41598-017-12953-9

**Published:** 2017-10-20

**Authors:** Shiqi Yang, Yongsheng Wang, Ruliang Liu, Aiping Zhang, Zhengli Yang

**Affiliations:** 1 Institute of Environment and Sustainable Development in Agriculture, Chinese Academy of Agricultural Sciences, Beijing, 100081 China; 2 Key Laboratory of Agro-Environment and Climate Change, Ministry of Agricultural, Beijing, 100081 China; 3 Key Laboratory of Regional Sustainable Development Modeling, Institute of Geographic Sciences and Natural Resources Research, Chinese Academy of Sciences, Beijing, 100101 China; 4 Institute of Agricultural Resources and Environment, Ningxia Academy of Agro-Forestry Science, Yinchuan, 750002 China

## Abstract

A five-year swine manure application trial and a study of nitrate leaching losses have been conducted. There were three treatments: traditional without manure (CK), traditional matched manure 4500 kg ha^−2^ (T1) and traditional matched manure 9000 kg ha^−2^ (T2). Nitrate nitrogen leaching losses at the 30-, 60-, and 90-cm soil layers were measured using the resin core method. The results indicate that the swine manure application did not noticeably increase soil nitrate leaching losses in the 30-cm layer. T1 (16.85 ± 0.40 kg ha^−2^) and T2 (17.01 °C0.46 kg ha^−2^) were not significantly different than CK (15.96 ± 0.41 kg ha^−2^) (P < 0.05), which was also the case at the 60-cm layer. However, there are significant differences between the treatments and CK at the 90-cm layer, although there were no significant differences between T1 and T2 in that layer. The application of manure can increase soil organic matter (SOM) and total nitrogen (TN). The SOM of T1 and T2 were increased to 0.95 g kg^−1^ and 1.41 g kg^−1^, respectively. The TN values of CK, T1 and T2 were 0.72, 0.78 and 0.88 g kg^−1^, respectively, in the 0 to 30 cm layer, and were improved by 7.72% and 22.04%.

## Introduction

Several lakes and rivers in China suffer from nitrogen and phosphate pollution. Twenty-four of 61 key lakes and pools have water quality below Class III. For the top ten rivers, 10.2% have inferior Class V water quality, 20.9% have Class IV–V quality, and 68.9% have Class I–III quality. Only 72.3% of drinking water sources in key cities attain the national water standard, and nearly 0.3 billion people in rural regions drink unsafe water^1^. TN, TP and COD from agricultural sources are approximately 2.7, 0.28, and 13.24 million tons, respectively, which accounts for 57.2%, 67.4% and 3.7%, respectively, of total emissions^2^. More than half of the groundwater in 14 investigated counties on the North China Plain have nitrate concentrations that exceed 10 mg L^−13^. Nitrate concentrations in 7.4% of the groundwater in Beijing suburbs exceed the national standard^4^.

The relationship between organic matter (OM) and soil nitrogen has always been of concern. Some researchers believe that OM can reduce nitrate loss^5,6^, but over-application can cause greater loss^7–9^. The inverse relationship between dissolved organic carbon (DOC) and NO_3_^−^ concentrations in Japanese streams is closely related to excess Nitrogen availability together with a carbon deficit in a soil environment^10^. To adjust the release of nitrogen into the soil, manure has replaced some chemical fertilizers^11^. To meet the N requirements of corn, poultry litter, which releases N more slowly compared to inorganic fertilizer, has been used; the use of poultry litter not only reduces N fertilizer costs for corn but also reduces the risk of nitrate N leaching into the groundwater^12^. Annual nitrate leaching losses in conventional plots are 4.4–5.6 times higher than in organic plots and integrated plots between conventional and organic plots, which demonstrates that organic and integrated fertilization practices more actively and efficiently support denitrifier communities, shift the balance of N emissions and nitrate losses, and cause less environmental harm^13^. Although only approximately 10% of the applied ^15^N-Iabeled fertilizer remained in the 0–30 cm layer of the control and plant manure plots, more than 25% of the applied ^15^N remained in the pig compost plot^14^. Mamo determined that nitrate leaching could be reduced by substituting compost for commercial inorganic fertilizer in vegetable fields^15^. A routinely cited environmental advantage of compost as a soil amendment is that it can reduce mineralization rates and decrease the potential for nitrate leaching by delaying the conversion of organic N to mobile and nitrate N^16^. Brinton reported potentially lower nitrate leaching from soils amended with composted rather than uncomposted manure in a maize field^17^. To reduce nitrate losses, organic fertilizers should not exceed 175 N kg ha^−2^ in nitrate vulnerable zones, but the index was 276 N kg ha^−2^ at the Rothamsted Experimental Station^18^. Bird guano resulted in greater nitrate losses when the application amount exceeded 11.2 t ha^−2^ ^19^.

In the Yellow River irrigation zone of Ningxia, the concentration of ammonium in the drainage channels is 20–30 mg l^−1^. Only 38.3% of the sections attain a Class V water standard, so at the outer-boundary section of the Yellow River, water quality is usually at Class V during the entire year. Nitrate concentrations exceed 10 mg l^−1^ for nearly half of the shallow groundwater^20^. The SOM is usually deficient; the values range from 9.2 to 14.5 g kg^−1^, and the average value is 10.2 g kg^−1^. The application rate of fertilizer N is as high as 301 kg ha^−2^, which is 1.6 times the national average. The total farmland nitrogen and nitrite levels are at 61–66% and 76–81%, respectively, in surface waters. Approximately 7 billion m^3^ y^−1^ of water is drawn from the Yellow River, and 2.5 billion m^3^ y^−1^ enters the Yellow River again by percolation and filtration from upper fields. The irrigation rate is 15–45 × 10^3^ m^3^ ha^−2^ for rice, 3.3–4.5 × 10^3^ m^3^ ha^−2^ for wheat/corn (intercropping), and 1.5 × 10^3^ m^3^ ha^−2^ for winter irrigation and spring irrigation^21^.

## Materials and Methods

### Study area

**Figure 1:**
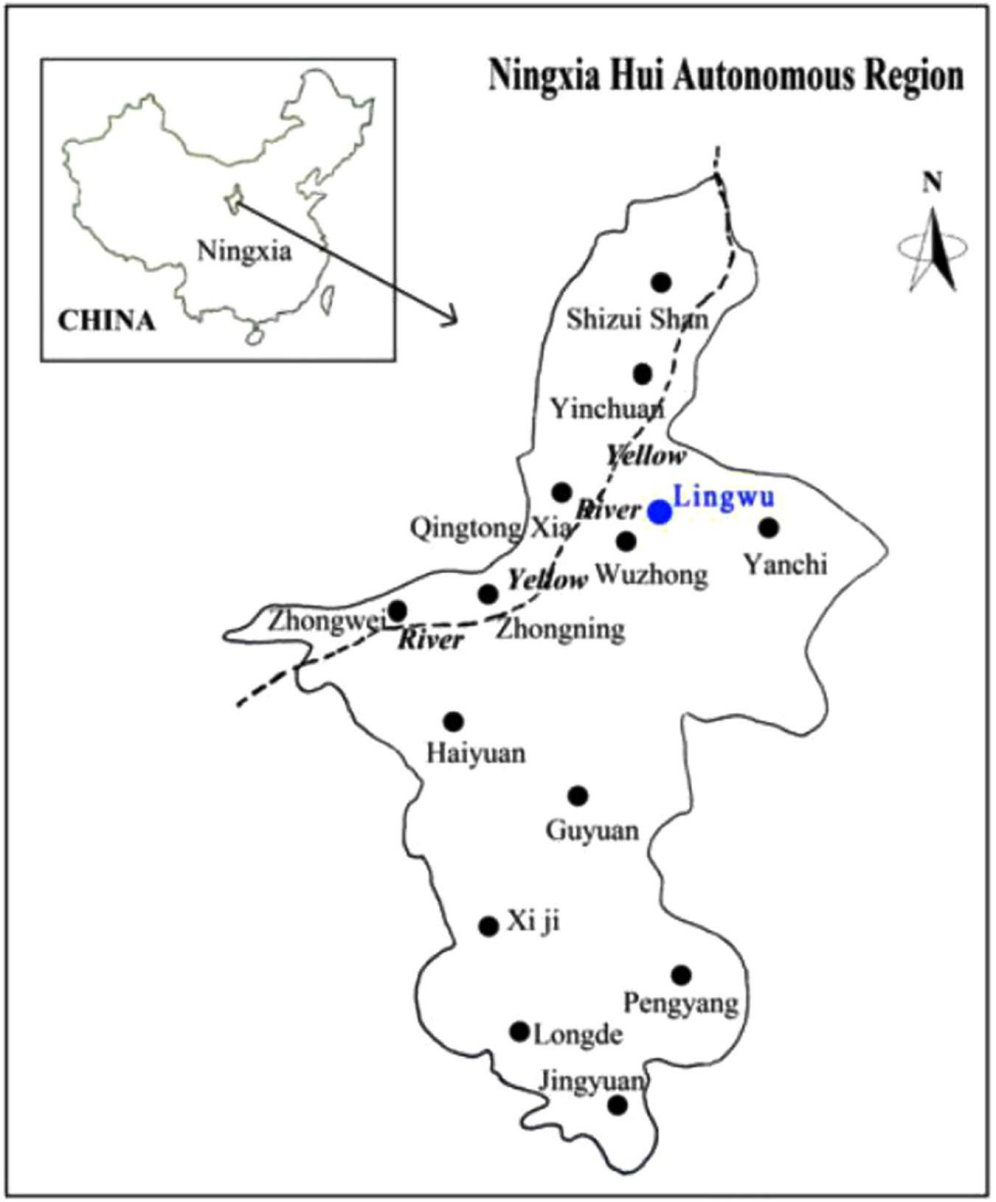
Location of the study area in the Yellow River irrigation area, North Ningxia. (Note: One of your co-authors created the figure with Photoshop, and had published the figure in another journal. From: Y Z Feng *et al*. Features and treatment of non-point source pollution in the Ningxia Yellow River area. African Journal of Agricultural Research. 6 (24), 5541–5550 (2011)).

The trial field (106°17′52″E, 38°07′26″N) is in Lingwu County in Ningxia, which is part of the upstream irrigation zone of the Yellow River (Fig. 1). The area is a temperate arid zone with low rainfall and a dry climate. The rainy season is from July to September and accounts for 70% of the total annual precipitation. Snows in the winter is rare. The average annual precipitation is only 193 mm yr^−1^ and the evaporation rate is 1,763 mm yr^−1^. There are approximately 150–164 frost free days, and the average annual temperature is 8.9 °C. The active accumulated temperature for temperatures ≥10 °C is 3,200–3,400 °C. The average elevation above sea level is 1,130 m. There are between 2,800 and 3,100 daylight hours in a year. Agriculture essentially relies on gravity flow systems. The soil type is classified as anthropogenic-alluvial with lower fertility (Table 1). The topsoil has a high salinity and a high pH. It is a single-harvest zone. The main crops are rice, corn and spring wheat, and the typical cropping system is rice-corn rotation.

**Table 1:** Physical and chemical properties of the trial field.

Soil depth (cm)	Bulk density (g cm^−3^)	SOM (g kg^−1^)	TN (g kg^−1^)	TP (g kg^−1^)	Soil available nutrients (mg kg^−1^)					Soil salt	pH
					N	P	K	NO_3_^−^N	NH_4_^+^−N		
00–30	1.49	13.78	0.87	0.80	93.43	27.34	145.87	10.21	0.98	1.13	8.59
30–60	1.58	7.33	0.75	0.78	63.76	14.89	137.10	6.97	0.96	0.91	8.76
60–90	1.43	4.14	0.49	0.50	34.17	4.42	98.25	6.23	0.93	0.74	9.21

### Experiment design

Each plot was insulated by brick walls with plastic film to prevent water interchange, 40 cm high above the ground and 80 cm deep beneath the soil. There were 3 treatments: traditional without manure (CK: 0 kg ha^−2^), swine manure application (T1: 4,500 kg ha^−2^) and swine manure application (T2: 9,000 kg ha^−2^). The plot area was 200 m^2^ with 3 replications. The field trials were conducted from 2009 to 2013. The crop was rice each year except for wheat in winter 2011. Swine manure was applied and plowed 0–25 cm in the topsoil before winter irrigation.

The rates of fertilizer application in the rice field were urea, 300 N kg ha^−2^, triple superphosphate, 105 P_2_0_5_ kg ha^−2^, and potassium chloride, 60 K_2_0 kg ha^−2^. Half of the nitrogen, total phosphate and potash were applied as base fertilizers, and the other 50% of the nitrogen was applied as a top dressing at a ratio of 3:1:1 at seeding (end of May), tillering (end of June), and booting (end of July) stages. The time of first irrigation in the paddy field was approximately mid-May, and the last irrigation was early July each year. The irrigation rate was 15, 000 m^3^ ha^−2^ in the rice growing period. Rice transplanting was in mid-May, and harvesting was in late September (approximately 120 days).

The fertilizer application rates in the wheat field were urea, 225 N kg ha^−2^, triple superphosphate, 150 P_2_0_5_ kg ha^−2^, and potassium chloride, 90 k_2_0 kg ha^−2^. Half of the nitrogen, total phosphate and potash were applied as base fertilizers. The remaining 50% of the nitrogen was applied as a top dressing at a ratio of 3:1:1 at seeding (early March), elongation (early May) and earing (early June) stages. The irrigation scheme included winter irrigation 1,350 m^3^ ha^−2^ (end of Oct.), greening, 900 m^3^ ha^−2^ (end of Mar.), elongation, 1050 m^3^ ha^−2^ (middle of May), and heading, 1,050 m^3^ ha^−2^ (early June). Sowing occurred on 4 Oct. 2010 and harvest occurred on 29 June of the following year (269 days later).

### Methods

**Figure 2:**
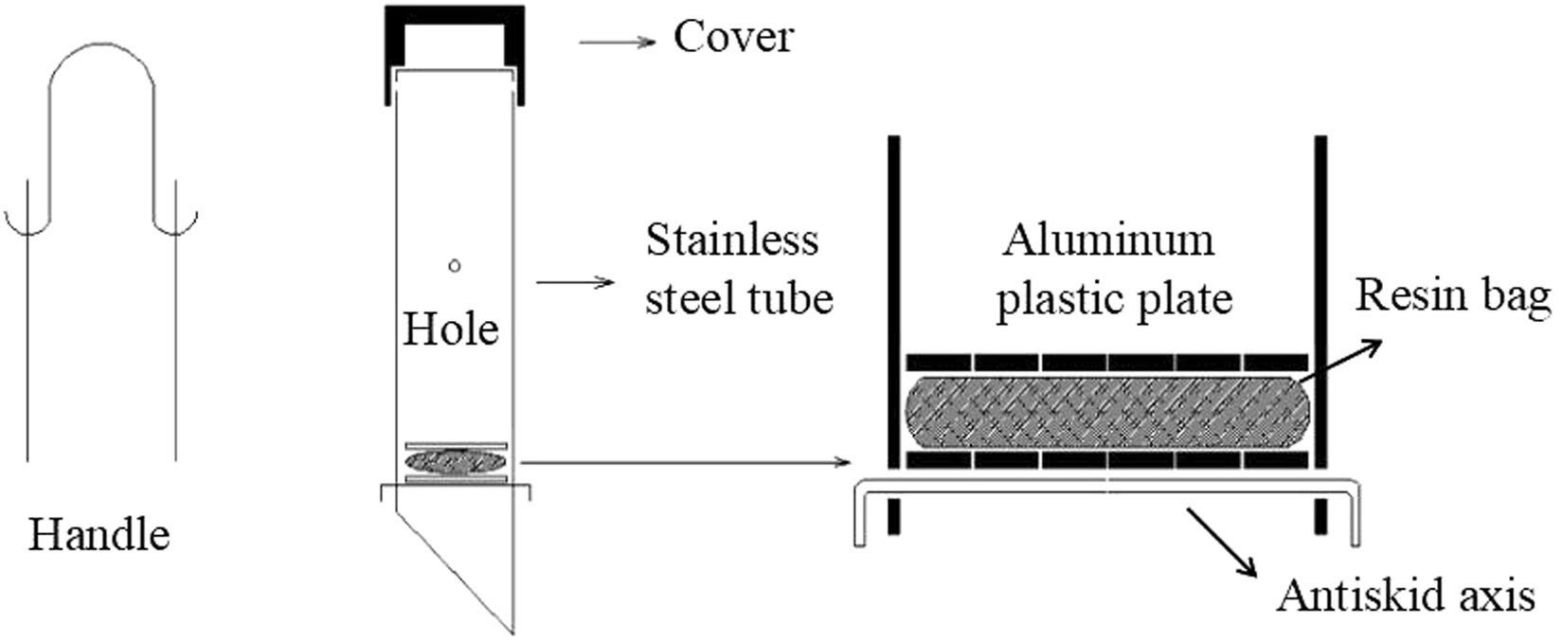
Resin core device used to determine soil nitrate loss.

Soil nitrate losses were determined using a resin-core device (Fig. 2, national patent No. 201020282864.4, 2010). This method had been used to determine soil nitrogen mineralization^22–25^. The resin-core device was comprised of a stainless steel pipe (diameter 76 mm × thickness 0.82 mm), resin bag (60 mesh nylon net, length 8 cm × width 8 cm), 2 pieces of aluminum plastic plate (diameter 74 mm with 13 eyes (diameter 3 mm)), cover, handle and antiskid axis. A strongly alkaline anion exchange resin (trade mark 717^#^) was used, and the pretreatment corresponded to the Method for Pretreating Ion Exchange Resins (GB/T 5476-1996). There were 4 holes in the pipe body for reducing the pressure difference between the inside and the outside of the pipe. The end of the pipe was a 10-cm long wedged surface for easy insertion into the soil. A 2-cm long aluminum plastic plate was above the wedge. The antiskid axis may have been fixed to prevent the aluminum plastic plates from dropping. The cover and the handle were convenient to place and draw the pipe body in the soil. There were 5 types of resin-core devices that were 42, 72, and 102 cm long. Initially, the resin-core devices were inserted just below the soil surface. Second, the pipes were lifted out, and the soil in the wedge and 2 cm above the wedge was collected. Third, the aluminum plastic plates, resin bags (16 g of resin in a bag), aluminum plastic plates, and antiskid axis were attached in sequence. Finally, the devices were placed back into the soil, and the wedge was refilled with soil. After a period of time, the devices were retrieved, the old resin bags were removed, and new resin bags were installed for the next test stage. The old resin bags were refrigerated. On each plot, 3 types of devices were placed in line and separated by 2 m, and the 3 duplications were placed diagonally along the plot.

The nitrate in the resin bags was extracted in Potassium chloride solution (1 mol/L KCl) and determined using ultraviolet spectrophotometry. The nitrate loss is calculated using the following equation:

$$nitrate\,loss\,(kg\,h{a}^{-2})=\frac{nitrate\,amount\,in\,resin\,(kg)}{cross\,sectional\,area\,of\,pipe\,({m}^{2})}\times 10,000\,({m}^{2})$$

The data were analyzed using SPSS 19 and Excel 2010, and the probability was determined to test the significance (α = 0.05) in different treatments using the one-way analysis of variance (ANOVA).

## Results

### Nitrate leaching loss

**Figure 3:**
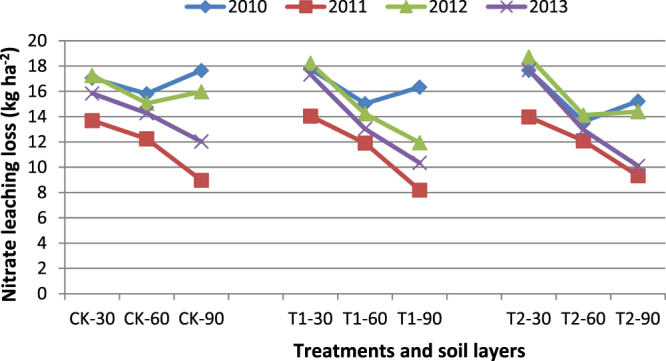
Nitrate leaching trends for 4 years.

Table 2 shows nitrate leaching losses. Nitrate leaching losses in the 30-cm layer were 16.95–17.50 kg ha^−2^ in the rice field and 13.91 kg ha^−2^ in the winter wheat field; there were significant differences (p < 0.05) between the rice and winter wheat fields. However, the treatments and CK were not significantly different (p > 0.05) in the 30-cm layer. In the 60-cm layer, T2 and CK were significantly different (p < 0.05) in 2010 and 2013. T1 and CK were significantly different (p > 0.05) only in 2013. T1 and T2 were significantly different (p < 0.05) only in 2010. In the 90-cm layer, T2 and CK, and T1 and CK were significantly different in 2010, 2012 and 2013. T2 and T1 were significantly different only in 2012. In the 90-cm layer, T2 and CK, and T1 and CK were all significantly different (p < 0.05) in 2010, 2012 and 2013. T2 and T1 were significantly different (p < 0.05) only in 2012. There were significant differences (p < 0.05) between the rice and winter wheat fields. The 90-cm layer and 30-cm layer, and the 6- cm layer and 30-cm layer were significantly different, but there were no significant differences between T1 and T2. Figure 3 shows the nitrate leaching trends. Nitrate leaching losses declined with increasing soil depth in all years, and the losses in the winter wheat field were less than in the rice field. Nitrate leaching losses in the rice field were very similar over 3 years, but the losses in the last year were less than in the first and third years.

**Table 2:** Nitrate leaching losses at different soil layers and treatments in the trial period.

Soil layers	Nitrate loss (kg ha^−2^)				Mean in the same soil layer (kg ha^−2^)	Notes
	2010	2011	2012	2013		
CK-30	17.05 ± 0.56a	13.69 ± 0.22a	17.26 ± 0.53a	15.84 ± 0.33a	15.96 ± 0.41a	Columns 2–4: significant differences between treatments; last column: significant differences of layers
T1-30	17.77 ± 0.67a	14.04 ± 0.17a	18.22 ± 0.37a	17.35 ± 0.39a	16.85 ± 0.40a	
T2-30	17.67 ± 0.34a	13.98 ± 0.42a	18.71 ± 0.85a	17.68 ± 0.21a	17.01 ± 0.46a	
mean	17.50 ± 0.52a	13.91 ± 0.27c	18.06 ± 0.58a	16.95 ± 0.31b	16.61 ± 0.42a	Significant differences of years
CK-60	15.81 ± 1.02a	12.25 ± 0.53a	15.06 ± 1.12a	14.28 ± 0.54a	14.35 ± 0.80a	Columns 2–4: significant differences between treatments; last column: significant differences of layers
T1-60	15.03 ± 0.90a	11.91 ± 0.75a	14.23 ± 1.85a	13.04 ± 1.01b	13.55 ± 1.13a	
T2-60	13.61 ± 1.44b	12.09 ± 0.80a	14.12 ± 2.33a	12.96 ± 1.22b	13.20 ± 1.45a	
mean	14.82 ± 1.12a	12.08 ± 0.69b	14.47 ± 1.77a	13.43 ± 0.92a	13.70 ± 1.13b	Significant differences of years
CK-90	17.65 ± 2.19a	8.97 ± 0.34a	15.97 ± 1.29a	12.03 ± 1.45a	13.66 ± 1.32a	Columns 2–4: significant differences between treatments; last column: significant differences of layers
T1–90	16.33 ± 1.38b	8.20 ± 0.91a	11.94 ± 2.11c	10.34 ± 1.64b	11.70 ± 1.51b	
T2-90	15.22 ± 0.79b	9.34 ± 0.79a	14.39 ± 2.03b	10.10 ± 1.31b	12.26 ± 1.23b	
mean	16.40 ± 1.45a	8.83 ± 0.68c	14.10 ± 1.81a	10.82 ± 1.47b	12.54 ± 1.35b	Significant differences of years
Mean of 3 layers	16.24 ± 1.03a	11.61 ± 0.55c	15.55 ± 1.39a	13.74 ± 0.90b	14.28 ± 0.97b	Significant differences of years

Notes: α = 0.05, a or b stand for significant differences in nitrate leaching losses.

### The process of soil nitrate leaching loss during the entire growth period

**Figure 4:**
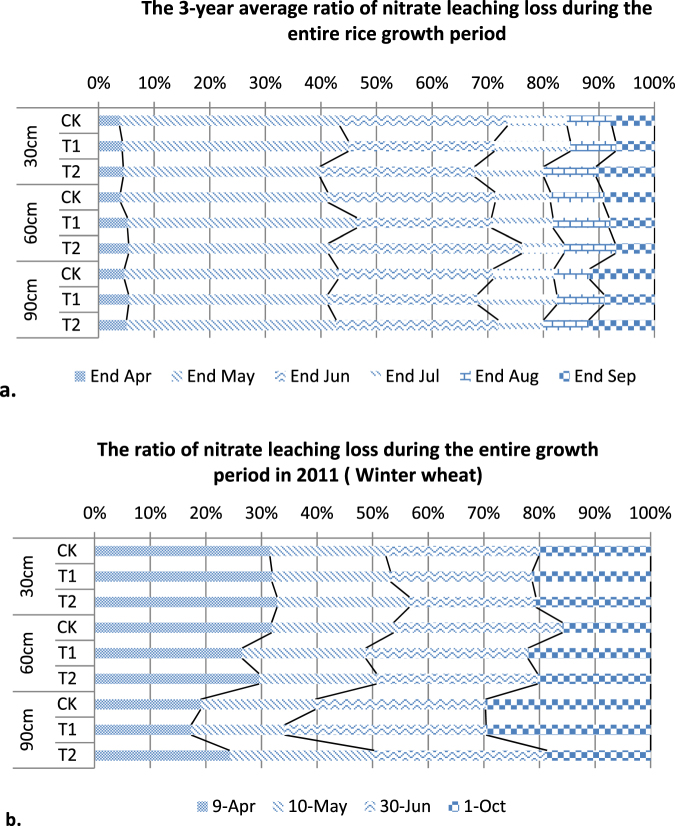
Ratio of nitrate leaching loss during the entire growth period.

In Fig. 4, the ratios of nitrate leaching losses are shown for different periods. In the rice field, the key period of soil nitrate leaching was before the end of June (45–50 days/130–140 growing days) (Fig. 4a); nearly 70% of nitrate had leached at the 30-cm layer, and almost 68–75% of nitrate had leached under the 30-cm layer. For winter wheat, there was a nearly 70–85% (Fig. 4b) nitrate leaching loss from each soil layer before the end of June. The key periods of soil nitrate leaching losses were at the early stages of rice and winter wheat; for rice it was at end of June (tillering phase), and for winter wheat it was at the end of June (earing phase).

## Discussion

### Soil nitrate concentration

As shown in Table 3, the soil nitrate concentration was low in the rice field in mid-April, increased at the end May and the end of June, and then gradually decreased at the end of July and August and early September. Soil nitrate concentrations were approximately the same in the middle of April and at the end of July (p > 0.05), at the end of May and the end of June, and at the end of August and early September. The difference between T2 and CK was significant except at the end of May and June, and the difference between T1 and CK was not significant in any month. The difference between T2 and T1 was significant, except at the end of May and June. Therefore, in the early growing period, the soil nitrate concentration was usually high and low, respectively, before rice transplanting and at the end of the growing period; furthermore, the soil nitrate concentration was approximately the same in the two stages. Therefore, the contribution of soil nitrate concentration from fertilizer N was greater than from swine manure. Of course, the contribution of nitrate from swine manure should not be ignored, especially in the irrigated field. Soil nitrate concentration in the winter wheat field at the end of May was greater than during other periods (p < 0.05), and it was lowest at the end of June. The difference between T2 and CK was significant in mid-April and at the end of May, and the difference between T1 and CK was significant only in mid-April. The difference between T2 and T1 was significant only at the end of May. Soil nitrate concentration in the winter wheat field was greater than in the rice field, possibly because there is no leaching in dry farming. No samples were obtained after harvest because weeds were difficult to eradicate in the winter wheat field.

**Table 3:** Soil nitrate concentration in the 30 cm layer (mg kg^−1^).

Crop	Mean	Date					
		Mid-Apr	End-May	End-Jun	End-Jul	End-Aug	Early Sep
Rice	CK	23.08 ± 3.35bB	35.43 ± 0.75 aA	35.73 ± 0.90 aA	24.78 ± 1.21aB	19.13 ± 1.83bC	20.15 ± 1.48bC
	T1	22.91 ± 1.07bC	34.52 ± 4.80 aA	34.56 ± 1.46 aA	25.93 ± 3.57aB	20.77 ± 2.03bD	20.64 ± 3.75bD
	T2	25.20 ± 1.97aB	35.46 ± 1.27 aA	35.45 ± 0.75 aA	23.72 ± 3.09bC	22.64 ± 3.25aC	22.24 ± 1.58aC
Winter wheat	CK	31.31 ± 1.41bB	37.16 ± 0.87bA	25.22 ± 1.11aC	—	—	—
	T1	34.32 ± 2.08aB	37.44 ± 1.34bA	25.07 ± 0.89aC	—	—	—
	T2	35.16 ± 1.97aB	41.31 ± 1.12 aA	24.92 ± 1.23aC	—	—	—

Notes: Small letters indicate statistical significance between different treatments during the same period, and capital letters indicate statistical significance between different periods for the same treatment. “−”indicates no samples.

Soil nitrate concentration dropped and the carbon to nitrogen ratio increased with increased organic matter; therefore, nitrate leaching can be controlled at a higher carbon to nitrogen ratio because soil microbes fix mineral nitrogen^26^. Too great a volume and long-term manure application possibly caused more nitrate to leach^27^. Soil nitrate accumulation increased as manure application increased, and manure over-application caused more nitrate to accumulate below a 200 cm depth^28^. Soil microbes convert nitrogen into nitrate and improve the probability of nitrate leaching under a high organic carbon to nitrogen ratio^29^. Soil nitrate concentration increased as soil depth increased, and the peak value appeared in the 100–120-cm layer in a peach orchard^30^; but Zhang found that the peak value of nitrate accumulation was in the 0–60-cm layer^31^. Fertilizer reduction, improved methods of fertilizing, and fertilizer efficiency can decrease soil nitrogen accumulation and leaching^32^. Manure over-application caused the nitrate concentration to increase sharply in the soil profile in a vegetable field^33^.

### Nitrate of soil leachate

There were 4 time samples of soil leachate in the rice field each year. Table 4 shows the soil leachate nitrate concentrations.

**Table 4:** Nitrate concentration of soil leachate (rice field) (mg l^−1^).

Soil layer	treatments	End-May	Early Jun	End-Jun	End-Jul
30 cm	CK	6.73 ± 0.54 aA	5.85 ± 0.64bA	4.53 ± 0.52bB	1.87 ± 0.26aC
	T1	6.95 ± 0.38 aA	6.72 ± 0.56bA	4.18 ± 0.27bB	1.81 ± 0.10aC
	T2	6.54 ± 0.51aB	7.92 ± 0.87 aA	6.01 ± 0.41aB	1.69 ± 0.34aC
60 cm	CK	4.30 ± 1.68cB	7.33 ± 0.34 aA	4.55 ± 0.39aB	1.99 ± 0.13aC
	T1	7.50 ± 0.83 aA	6.64 ± 0.17bA	5.39 ± 0.36aB	1.98 ± 0.31aC
	T2	6.23 ± 0.24bB	8.04 ± 0.35 aA	4.18 ± 1.09aC	2.21 ± 0.19aD
90 cm	CK	8.90 ± 0.96 aA	5.12 ± 0.39cB	3.66 ± 0.49bC	2.33 ± 0.21bD
	T1	7.04 ± 0.53bB	10.36 ± 1.06 aA	7.13 ± 2.94aB	2.31 ± 0.22bC
	T2	7.60 ± 1.14bA	7.74 ± 0.42bA	6.14 ± 0.25aB	4.70 ± 0.57aC

Notes: Small letters indicate statistical significance between different treatments during the same period, and capital letters indicate statistical significance between different periods for the same treatment. “—”indicates no samples.

In the 30-cm layer, the difference between T1 and CK reached significance in early and late June, as did the difference between T2 and T1 (p < 0.05). T1 and CK were not significantly different (p > 0.05) in any month. Nitrate concentration was not significantly different (p > 0.05) at the end of May compared with early June, but there were clear differences (p < 0.05) between early June, the end of June and the end of July. The sampling intervals were short, which led to similar nitrate concentrations at the end of May and in early June, and at the end of June, the nitrate concentration of soil leachate was not low, which was coincident with the soil nitrate concentration. At the end of July, the nitrate concentration of the soil leachate was greatly reduced. In the 60-cm layer, the difference between T2, T1 and CK reached significance at the end of May; the nitrate concentration of T1 was the largest. Only T1 and CK were significantly different in early June; T2 and CK were not significantly different, and CK was greater than T2. The treatments and CK were not significantly different at the end of June and July. The nitrate concentration differences in the 60-cm layer were greater than in the 30-cm layer between the end of May and early June. The nitrate concentration was reduced at the end of June and greatly reduced at the end of July. In contrast with the CK treatments, the differences in nitrate concentration in the 90-cm layer almost reaching significance in all periods, except T1 at the end of July, Almost all treatments were greater than CK. The change of nitrate concentration in the 90-cm layer was the same as in the 30-cm and 60-cm layers. The chicken manure application caused high soil leachate nitrate and ammonium concentrations in the paddy field^34^.

### Soil TN and SOM

In the 0–30-cm-layer, the TN values for CK, T1 and T2 were 0.72, 0.78 and 0.88 g kg^−1^, respectively, and were improved by 7.72% and 22.04% (P < 0.05), respectively. This result was the same as for the other tests. In the 30–60-cm layer, the TN values of CK, T1 and T2 were 0.42, 0.41 and 0.45 g kg^−1^, respectively and were improved by −1.26% and 6.01% (P > 0.05), respectively. In the 60–90-cm layer, the TN values of CK, T1 and T2 were 0.27, 0.32 and 0.31 g kg^−1^, respectively, and were improved by 21.39% and 16.46% (P < 0.05), respectively. The results show that soil nitrogen leached from the topsoil into the deep layer and finally into the groundwater and that nitrogen losses increased with nitrogen application. Contrasted with a CK of 12.67 g kg^−1^ in the 0–30-cm layer, the SOM of T1 and T2 were increased to 0.95 g kg^−1^ and 1.41 g kg^−1^, respectively, in 2013. Therefore, swine manure application can increase soil organic matter; most of the tests support the results. Manure application can increase organic carbon in the plow layer and plow pan (P < 0.05); TN, TP, TK and available nutrients were clearly increased. Soil porosity increased by 11%, and the water stability of soil aggregates was stronger than CK. However, the tensile strength of the soil was clearly reduced. MBC and MBN were 2.1 and 1.5 times greater than CK in the plow layer^35^.

### Cropping patterns and soil nitrate leaching loss

Soil nitrogen leaching losses were affected by cropping patterns, which include the crops and the cropping system. Nitrate leaching losses in the paddy field were greater than in the winter wheat field; the primary reason was the presence and quantity of irrigation. Nitrate leaching losses increased with increased irrigation. There was greater nitrate leaching loss in the seedling stage because of the immature crop roots; soil nitrate could not be absorbed more rapidly and completely than required in the seeding stage. A new fertilizing technique was named “the technique of fertilizer nitrogen backward”, which means that the fertilizer rate is reduced in the early stage and increased in the middle stage so that the total dose is not changed^36^. The top 4 out of 12 cropping patterns, which were winter wheat-rice, spring wheat-Chinese cabbage, winter wheat-silage corn and winter wheat-oil sunflower, clearly reduced the residual soil nitrate, so repeated cropping and intercropping reduced nitrate leaching losses and protected shallow groundwater^37^. The traditional cropping system has a long fallow from early October to early March or mid-May (150 or 220 days), and the nitrate leaching losses were purely nitrogen (0.69–0.81 kg ha^−2^)^38^. Nitrate leaching losses at the vegetable farm were greater than at the crop farm because of the fertilization rate. In agroforestry systems, tree roots can absorb soil N in the deep soil and reduce the amount of nitrogen that leaches into the groundwater^39^. The nitrogen peak was in the spring formative period for forestry and was in the grain formative period for crops^40^. For cereal crops, application of 200 kg ha^−2^ of nitrogen in one season should be considered, and the application of organic and inorganic fertilizer for the wheat-corn rotation should not exceed 400 kg ha^−2^ (12,000 kg ha^−2^ yield) on the North China Plain^41^.

### The shallow groundwater and soil nitrate leaching loss

Nitrate leaching losses in the 90-cm layer have a greater chance of migrating into water because the groundwater level is only 120–200 cm high during the planting season. The amount of leakage water was nearly 100–120 cm in the paddy field each year, and the amount of nitrate lost through leaching losses was 8.00–8.69 kg ha^−2^ y^−1^ of pure nitrogen. If the leakage water and nitrate lost to leaching enter the groundwater together, the nitrogen concentration in the leachate would be 0.67–0.87 mg l^−1^; the loss was almost equal to 2.96–3.85 mg l^−1^ for nitrate leachate. The nitrate concentration exceeded 10 mg l^−1^ in some places, and it was greater in the planting season. Zhao demonstrated that nitrate and organic nitrogen concentrations in groundwater were 11.85–26.12 and 0.64–5.89 mg l^−1^, respectively, under greenhouse vegetables and 0.12–4.97 and 0.03–1.00 mg l^−1^, respectively, under farmland in the Ningxia irrigation zone^42^. The dominant N leaching form was nitrate (accounting for 57.3–92.0% of TN), and the secondary form was dissolved organic nitrogen (7.8–42.5%); only approximately 1% of nitrite was leached^42^. Ju noted that greenhouse vegetable farming caused soil nitrate accumulation and leaching; the groundwater nitrate concentration was 9–274 mg l^−1^ and the soil nitrate concentration was 270–5038 kg ha^−2^ in the 0–90-cm soil layer in Huimin County, Shandong Province, where nitrate concentration in 99% of the groundwater was more than 10 mg l^−1^ ^43^. Song analyzed soil nitrate leaching losses of greenhouse vegetables that reached 152–347 kg ha^−2^ in the 0–100-cm soil layer in Shouguang County, Shandong Province, which was closely related to groundwater pollution^44^. Nitrate in groundwater was demonstrated to come from farmland soil because of large applications of fertilizer nitrogen^45^.

## Conclusions

Manure application at 6,000 to 9,000 kg ha^−2^ does not cause an obvious increase or decrease in soil nitrate leaching. Therefore, in the Ningxia irrigation zone, some type of manure application should be practiced as this study found that rice yield is increased from 12.26% to 11.55%, and the winter wheat yield is increased from 9.32% to 12.52%. In addition, the application of manure makes it possible to reduce the use of nitrogen fertilizer, crop yields do not drop, and soil nitrate leaching losses do not increase.
